# The inhibitory effect of a *Platycodon* root extract on ultraviolet B-induced pigmentation due to a decrease in Kit expression

**DOI:** 10.1007/s11418-014-0836-x

**Published:** 2014-05-06

**Authors:** Shinya Kasamatsu, Akira Hachiya, Yoshie Shimotoyodome, Akiyo Kameyama, Yuki Miyauchi, Kazuhiko Higuchi, Taketoshi Fujimori, Atsushi Ohuchi, Yusuke Shibuya, Takashi Kitahara

**Affiliations:** Biological Science Laboratories, Kao Corporation, Haga, Tochigi 321-3497 Japan

**Keywords:** Skin pigmentation, Kit, *Platycodon*, Stem cell factor, UVB

## Abstract

The signaling of stem cell factor (SCF) through its receptor Kit is known to play an important role in regulating cutaneous melanogenesis. In the course of UVB-induced pigmentation, the expression of membrane-bound SCF by epidermal keratinocytes is upregulated at an early phase and subsequently activates neighboring melanocytes via their Kit receptors. In order to identify effective skin-lightening materials, we screened botanical extracts to determine their abilities to diminish Kit expression in melanocytes. A *Platycodon* root extract was consequently found to have a remarkable inhibitory activity on Kit expression. When the extract was applied to three-dimensional human skin substitutes in vitro and to human skin in vivo after UVB irradiation, their pigmentation was significantly reduced, confirming the substantial contribution of the suppression of SCF/Kit signaling to preventing or inhibiting melanin synthesis. These data demonstrate that a *Platycodon* root extract is a promising material for a skin-lightening product to improve pigmentation-related diseases.

## Introduction

Pigmentation plays an intrinsic role in protecting the skin from UV-induced photo damage, such as DNA damage [1]. However, aberrant pigmentation is considered to be a source of significant psychosocial distress all over the world. Especially in Asia, many people pay attention to technologies and related products that can lighten their skin color.

In the course of UVB-induced pigmentation, melanocytes in the basal layer of the epidermis are stimulated to proliferate, followed by the enhanced production and activation of tyrosinase, the rate-limiting enzyme required for melanin synthesis [2]. Several cytokines and chemokines, such as stem cell factor (SCF), endothelin-1 (ET-1), α-melanocyte-stimulating hormone (α-MSH), histamine and prostaglandin E2, have been shown to be released from epidermal keratinocytes in response to UVB exposure [3, 4]. Among them, we have focused on the significant role of SCF in UVB-induced melanogenesis and have reported that UVB irradiation augments the expression of membrane-bound SCF in epidermal keratinocytes to activate neighboring melanocytes via Kit, the SCF receptor. Kit is a receptor tyrosine kinase mainly composed of five extracellular immunoglobulin-like domains, a transmembrane domain and two intracellular tyrosine kinase domains. After the binding of SCF to the Kit extracellular domain, Kit is dimerized and then proximally two kinase domains within the dimer *trans*-phosphorylate reciprocal intracellular domains, resulting in the activation of melanocytes through various signaling cascades [5]. With regard to the important role of Kit in UVB-induced melanogenesis, the injection of a Kit-inhibitory antibody abolishes UVB-induced pigmentation on the dorsal skin of pigmented guinea pigs [4, 6], illustrating the pivotal role of SCF/Kit signaling in UVB-induced pigmentation.

The findings above led us to hypothesize that the inhibition of Kit expression would more efficiently lighten human skin, and we initiated a screening to identify botanical extracts with inhibitory activities on Kit expression in human melanocytes. In the present study, we report that a *Platycodon* root (*Campanulaceae*) possesses the ability to suppress Kit expression in a dose-dependent manner in melanocytes and significantly reduces melanogenesis in three-dimensional human skin substitutes (HSSs) in vitro and in UVB-induced pigmentation of human skin in vivo.

## Results and discussion

**Fig. 1:**
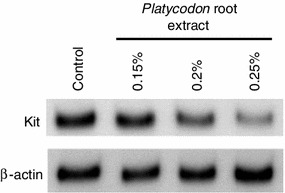
NHEMs were treated with 0.15, 0.2 or 0.25 % (v/v) *Platycodon* root extract for 5 days. Cells were solubilized and used in Western blotting to examine the expression levels of Kit normalized against β-actin. Each band shows a representative of similar results, which were repeated three times

**Fig. 2:**
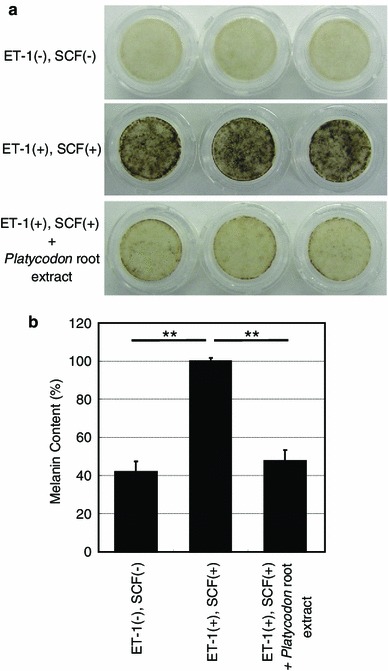
Three-dimensional HSSs were cultured with or without 0.4 % (v/v) *Platycodon* root extract in the presence of ET-1 and SCF for 14 days. **a** Photographs demonstrate the remarkable inhibitory effect of this extract on the ET-1/SCF-stimulated melanin synthesis. **b** Melanin content of solubilized HSSs was measured using an absorbance meter. The values reported are the mean ± SD from three samples in each group. **P < 0.01

**Fig. 3:**
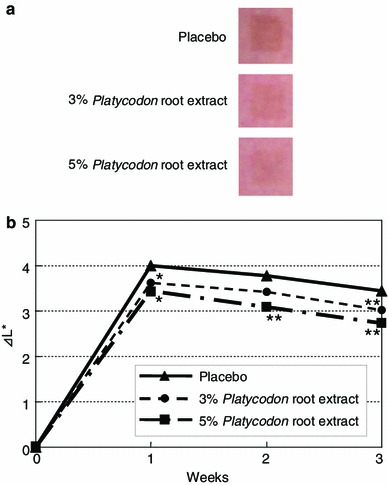
The upper arms of ten volunteers were irradiated with UVB exposure at 2 MED. After the irradiation, the irradiated areas were topically treated with 3.0 or 5.0 % (v/v) *Platycodon* root extract-containing lotion or with a placebo lotion twice a day for 3 weeks. **a** Photographs at 3 weeks after the irradiation demonstrate the remarkable inhibitory effect of this extract on UVB-induced pigmentation in a dose-dependent manner. **b** The intensities of pigmentation were measured using a color difference meter. The efficacy of the extract is expressed as the delta L* value compared to that of a placebo lotion. *P < 0.05; **P < 0.01 versus placebo control

We screened a number of botanical extracts for their abilities to inhibit Kit expression in normal human epidermal melanocytes (NHEMs). Western blotting analyses consequently showed that the *Platycodon* root extract substantially reduces Kit expression in a dose-dependent manner (Fig. 1). In order to determine its inhibitory activity on melanogenesis in vitro, its efficacy against melanin synthesis was examined in three-dimensional HSSs treated with ET-1 and SCF since the synergistic impact among them on the phosphorylation of mitogen-activated protein kinase (MAPK) and Kit has been documented [7]. They have biphasic roles in the course of UVB-induced skin pigmentation in vivo where SCF expression is stimulated at the early phase followed by the augmentation of ET-1 expression concomitant with increased tyrosinase expression [4]. After confirmation of the synergistic stimulation of ET-1 and SCF on melanin synthesis in HSSs (Fig. 2) as well as the negligible contribution of α-MSH (data not shown), a remarkable suppression of melanin synthesis compared to the control without extract was observed following addition of the *Platycodon* root extract. Additionally, it was also confirmed that cell viability was not changed by the application of this extract to HSSs (data not shown). This inhibitory effect on melanin synthesis in HSSs was shown to be equal to that of pifithrin-α, the inhibitor of SCF-inducing phosphorylation of MAPK, as we previously reported [8]. Additionally, our clinical study revealed that application of this extract significantly depresses UVB-induced pigmentation even at 1 week after irradiation in a dose-dependent manner (Fig. 3). This inhibitory efficacy of 5 % *Platicodon* root extract on UVB-induced pigmentation was found to be comparable to that of the 0.5 % extract of *M. Chamomilla,* the inhibitor of ET-1-mediating pigmentation, when their delta L* values were relatively compared to their placebo controls after 3-week application [9]. These results confirm the effect of decreasing Kit expression on the prevention or inhibition of skin pigmentation in vivo, especially after UVB exposure.

It is of interest to consider where this material with the pigment lightening activity might be effectively utilized apart from reducing UVB-induced pigmentation. It has been reported that the signaling cross-talk between ET-1 and SCF plays a role in regulating melanin synthesis in human melanocytes [7]. Additionally, increases in their expression have also been documented in senile lentigos (age spots) [10, 11]. Considering the markedly inhibitory effect of the *Platycodon* root extract on melanogenesis in HSSs in the presence of ET-1 and SCF, this Kit expression-inhibiting extract might be used to effectively lighten age spots, too. In the near future, examination to clarify the efficacy of this extract on age spots needs to be done.

In conclusion, the present study demonstrates that a *Platycodon* root extract effectively inhibits both ET-1- and SCF-induced pigmentation in three-dimensional HSSs and in UVB-induced pigmentation of human skin via the inhibition of Kit expression, suggesting that this extract is a promising material for an effective skin-lightening product.

## Experimental section

### Materials

Normal human epidermal melanocytes and three-dimensional HSSs (MEL-300A) were purchased from Kurabo Corp. (Osaka, Japan). Human recombinant SCF and anti-Kit-specific antibody were obtained from Immuno-Biological Laboratories Co. (Gunma, Japan). Human recombinant ET-1 and anti-β-actin-specific antibody were obtained from Sigma-Aldrich Co. (St Louis, MO, USA). Other chemicals were of reagent grade. *Platycodon* root (Lot No. CT-170502) were purchased from Shinwa Bussan Co., Ltd. (Osaka, Japan). The roots were cultivated in Haozhou of the Anhui Province, China, and were harvested in 2004. The verification test of *Platycodon* root was performed based on the standard test of the Japanese Pharmacopoeia. For future reference, a voucher of the sample has been deposited at the Kao Biological Science Laboratories (No. 050609).

### Preparation of the *Platycodon* root extract

The sliced and dried samples of *Platycodon* root (1.0 kg) were extracted twice with 90 % ethanol (10 l) at room temperature for 7 days. The resulting extract was filtered and concentrated under reduced pressure. The residue (96 g) was dissolved with 50 % ethanol and filtered through no. 2 Advantec filter paper. The residue weight percentage of this extract was about 2.5 (weight/volume). HPLC analysis of *Platycodon* root extract had been previously performed, resulting in identification of the structure of major saponins such as platycodin A, C and D [12].

### Cell culture

Normal human epidermal melanocytes were maintained in medium 254 (Kurabo Corp.) supplemented with 5 μg/ml insulin, 5 μg/ml transferrin, 3 ng/ml human recombinant fibroblast growth factor (rFGF), 0.18 μg/ml hydrocortisone, 3 μg/ml heparin, 10 ng/ml phorbol 12-myristate-13-acetate, 0.2 % (v/v) bovine pituitary extract (BPE) and 0.5 % (v/v) fetal bovine serum (FBS) at 37 °C with 5 % CO_2_, as previously described [13].

### Western blotting analysis

The expression levels of Kit and β-actin in NHEMs treated with the *Platycodon* root extract were analyzed using Western blotting. Sub-confluent NHEMs in 2.5-cm-diameter dishes were cultured in conditioned medium [Medium 254 supplemented with 5 μg/ml insulin, 5 μg/ml transferrin, 3 ng/ml human rFGF, 0.18 μg/ml hydrocortisone, 3 μg/ml heparin, 0.2 % (v/v) BPE and 0.5 % (v/v) FBS] for 3 days. Followed by the culture in the conditioned media in the presence of the *Platycodon* root extract [0, 0.15, 0.2 and 0.25 % (v/v)] for 5 days, cells were solubilized in cell lysis buffer (Cell Signaling Technology, Danvers, MA, USA) supplemented with 1 mM phenylmethylsulfonyl fluoride (Sigma-Aldrich Co.). After 5 μg protein was separated on 7.5 % SDS gels (Bio-Rad Laboratories, Hercules, CA, USA), they were transferred to Sequi-Blot PVDF membranes (Bio-Rad Laboratories) and incubated with a polyclonal rabbit anti-Kit-specific antibody or with a monoclonal mouse anti-β-actin-specific antibody followed by incubation with appropriate secondary antibodies corresponding to rabbit or mouse IgG (GE Healthcare UK Ltd., Buckinghamshire, UK). Subsequent visualization of antibody recognition was performed using Enhanced ChemiLuminescence Plus (GE Healthcare UK, Ltd.) according to the manufacturer’s instructions.

### Measurement of melanin content in three-dimensional HSSs

According to the manufacturer’s instructions, three-dimensional HSSs were maintained in EPI-100MM-113 medium (Kurabo Corp.) at 37 °C with 5 % CO_2_. Human skin substitutes were incubated with ET-1 and SCF (10 nM each) in the presence or absence of 0.4 % (v/v) *Platycodon* root extract for 14 days. Human skin substitutes were washed three times with phosphate-buffered saline and once with diethyl ether mixed with three volumes of ethanol. Human skin substitutes were then washed one time with diethyl ether and incubated at 50 °C for 2 h until dry, followed by solubilization in 200 μl 2 M NaOH for the measurement of melanin content using an absorbance meter (Microplate Reader model 550; Bio-Rad) at 405 nm and a melanin standard (Sigma-Aldrich Co.).

Evaluation of the inhibitory efficacy of the *Platycodon* root extract on human skin pigmentation.

UVB irradiations were performed using UVB lamps (Toshiba SE lamps) on the upper arms of ten healthy Japanese volunteers as described elsewhere [4]. After UVB irradiation at two minimal erythema doses, the irradiated areas were treated topically with a 3.0 or 5.0 % (v/v) *Platycodon* root extract-containing lotion or a placebo lotion twice a day for 3 weeks. At the start of the study and at 1, 2 and 3 weeks after the irradiation, the intensities of the induced pigmentation were measured as previously described [4]. This study was approved by the Ethical Committee of the Kao Biological Science Laboratories in accordance with the Declaration of Helsinki, and informed consent was obtained from each volunteer before the commencement of the study.

## Statistics

The level of significance of differences in melanin contents of HSSs or in delta L* values of skin color was calculated using Tukey’s test or the paired Student’s *t* test, respectively. Differences in the mean or raw values among treatment groups were considered significant when *P* < 0.05.
